# Adaptive Notch Filter for Piezo-Actuated Nanopositioning System via Position and Online Estimate Dual-Mode

**DOI:** 10.3390/mi12121525

**Published:** 2021-12-08

**Authors:** Chengsi Huang, Hongcheng Li

**Affiliations:** School of Electromechanical Engineering, Guangdong University of Technology, Guangzhou 510006, China; huang184280@163.com

**Keywords:** online system identification, adaptive notch filter, variable forgetting factor, recursive least squares algorithm, piezoelectric actuator, nanopositioning

## Abstract

Due to the excellent advantages of high speed, high precision, and driving force, piezoelectric actuators nanopositioning systems have been widely used in various micro/nanomachining fields. However, the inherent resonance dynamic of the nanopositioning system generated by the flexure-hinge greatly deteriorates the positioning performance and limits the closed-loop bandwidth. Even worse, the notch filter for eliminating the effect of resonance does not work due to the varying resonant frequency resulting from the external disturbance or mass load. To this end, an adaptive notch filter for piezo-actuated nanopositioning system via position and online estimate dual-mode (POEDM) has been proposed in this paper, which can estimate the varying resonant frequency in real-time and suppress the resonance to improve the closed-loop bandwidth. First, a novel variable forgetting factor recursive least squares (VFF-RLS) algorithm for estimating resonant frequency online is presented, which is robust to the noise and provides the performances of both fast tracking and stability. Then, a POEDM method is proposed to achieve the online identification of the resonant frequency in the presence of noise and disturbance. Finally, a series of validation simulations are carried out, and the results indicate that, the frequency of input signal and the bandwidth have been achieved up to 12.5% and 87.5% of the first resonant frequency, respectively.

## 1. Introduction

Piezo-actuated (PZT) nanopositioning system refers to flexure-hinge-guided mechanisms driven by piezoelectric actuator, which has attracted a wide range of researchers’ interest. Compared with the traditional servo system, PZT nanopositioning system has the advantages of high speed, high precision and driving force [1]. The atomic force microscope, developed based on PZT, makes a significant contribution in the fields such as bionanotechnology, material science, and nanomachining [2]. To cater for the requirement of high precision, the PZT nanopositioning tools, which achieve the degree of nanoscale or even sub-nanoscale positioning accuracy, are widely used in positioning of wafers and mask alignment [3,4]. Thereby, PZT nanopositioning system plays a key role in position technology and has a wide range of applications. However, these applications pose challenges in terms of control, where one of the greatest is the vibration induced by lightly damped resonance [5,6]. Due to the vibration, the gain margin of the system is reduced severely, which limits the bandwidth. As a result, it is difficult to employ a high gain feedback controller to obtain good performance of the system [7]. In practical applications, the frequency of input signal is often restricted to between 1% and 10% of the first resonant frequency of PZT [8,9].

Aiming at the issue of vibration, various control approaches have been proposed. An effective method is position feedback control, which achieves the high gain controller via feedforward compensation or inner position loop to dampen the resonance, including delay position feedback [5], positive position feedback [8], and recursive position feedback [9]. Besides, a Notch filter (NF) has been demonstrated as a simple and effective approach to suppress the resonance [10,11]. The resonant component can be filtered by inverting the resonance to improve the bandwidth. However, it is commonly impossible but desirable to load mass in many applications, which will lead to the resonant frequency shifts [12]. If the frequency shifts by only 1%, the system becomes unstable when the high gain controller or NF is used. Fortunately, adaptive notch filter (ANF) can be adopted as an effective approach for the time-varying resonant frequency, where the center frequency of ANF can be updated in real-time [13]. ANF plays an important role in signal processing [14,15] as well as other applications, such as launch vehicles [16], and hard disks [17]. Moreover, there are two main problems that prevent the use of ANF in the PZT nanopositioning system. One of the problems is that it is hard to carry out online system identification in a closed-loop system. The available information of the closed-loop system, which is obtained directly by measurement, is much less than that of the open-loop system. Furthermore, due to the feedback path, the disturbance exists in the entire loop of the closed-loop system. As a result, they generate an incorrect relationship between the input signal and itself [18], which leads to an increase in the union error of the online estimation. Another difficulty is that the performance of online system identification algorithms is sensitive to the noise, whose object is to make the predict error tend to be zero via the VFF-RLS algorithm. As the function of variable forgetting factor (VFF) is defined as being associated with the predict error included the noise, the fluctuation of online estimation is generated, which reduces the performance of the algorithm. In addition, the purpose of using VFF is to overcome the data saturation phenomenon of RLS algorithm, modify the parameters of the reference model effectively and ensure the stability [19,20]. Therefore, it is necessary to develop a VFF that is robust to noise and easy to be implemented.

This paper aims to estimate the varying resonant frequency in real-time and improve the closed-loop bandwidth of the PZT nanopositioning system. Herein, an ANF for nanopositioning system via POEDM has been presented. As far as known, this work is the first attempt to introduce online system identification via dual-mode method into the field of PZT nanopositioning. The contributions of this work can be summarized as follows:

1. A novel VFF-RLS algorithm based on absolute mean error has been proposed. The VFF varies according to the relative error boundary, which is robust to the noise. It is easy to implement parameters turning and provide good performances of both fast tracking and stability.
2. A POEDM method has been developed. The benefit of POEDM is that it combines the open-loop system identification with closed-loop feedback control via a simple structure. By this achievement, it is easy to utilize online estimation of PZT nanopositioning system in real-time to obtain good tracking performance.

This article is organized as follows. The modeling and problems of PZT are described in Section 2. In Section 3 and Section 4, the design and analysis of novel VFF-RLS algorithm and POEDM method have been presented, respectively. A series of simulations in terms of the resonant frequency online estimate and tracking performance have been carried out in Section 5, which aims to verify the effectiveness of VFF-RLS algorithm and POEDM, respectively. The conclusions are given in Section 6.

## 2. Modeling and Problem Statement

### 2.1. Modeling of Piezo-Actuated Nanopositioning System

First, the reference model of PZT is required to be determined for online estimation. In terms of control, the characteristics of PZT can be divided into two behaviors. One of them is hysteresis, the other is the dynamics consist of creep and vibration [21]. By employing high gain controller and high-speed motion, the effect of creep and hysteresis can be reduced, respectively [22]. However, the greatest limitation of the above approaches is the low gain margin caused by the vibration. Therefore, when it is assumed to work in the linear area, as well as utilizing the high gain controller and high-speed motion, PZT can be modeled only considering the effect of vibration to study. According to the frequency response of PZT obtained in these papers [9,22,23,24], it can be noted that there is a sharp resonant peak at the first resonant frequency. Due to the sharp peak, the gain margin of PZT becomes low and places at −20 to −10 dB commonly. Moreover, this peak is caused by the issue of vibration. To this end, PZT can be characterized as being closed to a unity-gain second-order low-pass system as follows:

(1) $$G(s)=\frac{\omega_{s}^{2}}{s^{2}+2\cdot \xi \cdot \omega_{s}\cdot s+\omega_{s}^{2}},$$

where $\omega_{s}$ and ξ are the resonant frequency and damping ratio of PZT, respectively.

### 2.2. Description of the PZT Control Problem

**Figure 1:**
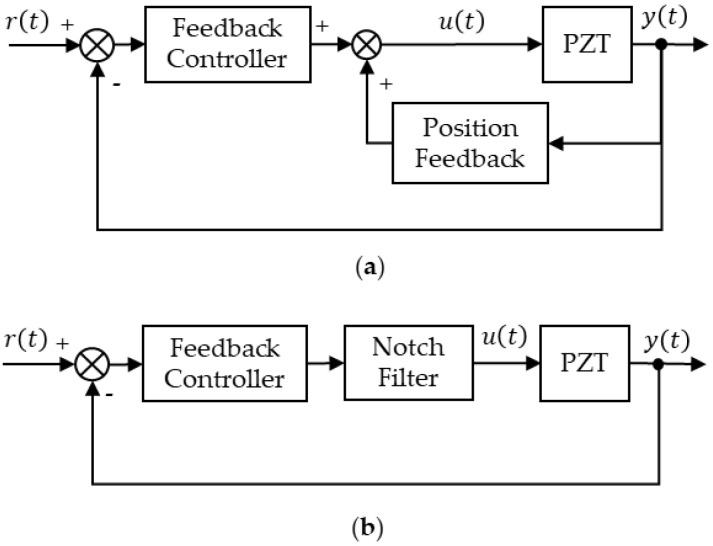
Block diagram of: (**a**) position feedback control; (**b**) notch filter.

To meet the requirements of high precision, fast response and high resolution, feedback control must be employed in the PZT nanopositioning system. In order to deal with the effects of sharp resonant peak in (1) as well as easily implement, the following methods are often used:

1. Position feedback (PF) control, as a type of damping control, increases the damping ratio of system to improve the gain margin via an inner position loop as shown in Figure 1a. Thus, a high gain controller can be used to provide good tracking performance. In addition, since only the position feedback is required, it is easy to be utilized.
2. By using NF to eliminate the resonant component in input signal of PZT, the block diagram is shown in Figure 1b. It is designed via inverse model, which is simple to analyze and offers excellent closed-loop bandwidth. By employing NF, the bandwidth is up to or even greater than the resonant frequency [10].

However, the varying resonant frequency caused by the applications of mass load deteriorates the performances of the above approaches greatly. Because it is designed by inversion techniques, the major problem of NF is sensitive to the frequency, which will cause the system to become unstable. Although PF control is insensitive to the frequency, it provides a lower bandwidth than NF when matching the resonant frequency. The above analysis will be demonstrated in Section 5. Thereby, NF and PF control are not effective methods to deal with the time-varying resonant frequency.

### 2.3. Description of the Online Frequency Estimate Problem

As described in Section 1, ANF is an effective approach to solve the problem of varying resonant frequency, where the central frequency can be updated in real-time via online estimation. The transfer function of ANF is similar to NF, and can be illustrated as follows:

(2) $$H(s)=\frac{s^{2}+\omega_{n}^{2}}{s^{2}+2\cdot \xi_{n}\cdot \omega_{n}\cdot s+\omega_{n}^{2}},$$

where $\omega_{n}$ and $\xi_{n}$ are the central frequency and damping ratio of ANF, respectively. The greatest key to utilizing ANF is the implementation of central frequency update in real-time, where the $\omega_{n}$ is replaced by a variable $\omega_{n}(t)$ to match the $\omega_{s}$. Therefore, the resonant frequency must be estimated in real-time.

Aims at estimating the resonant frequency in real-time, online system identification should be employed. Closed-loop online system identification refers to estimating the parameters of closed-loop system without changing the system to open-loop, which provides better performances than open-loop. Except for the disturbances problem of online estimation introduced in Section 1, the same output of a stable closed-loop system under the input signals with different feedback effects also hinders the use of closed-loop online system identification. These issues lead to increased union error or even unstable online estimation [25]. According to the system identification theory, in order to ensure the successful implementation of closed-loop online system identification, at least one of the conditions from a1 to a3 must be satisfied. Meanwhile, the condition b also needs to be met:

a1. The order of controller must be high enough;
a2. A nonlinear or time-varying controller is employed;
a3. A large enough delay unit exists in either the forward or feedback path.
b. The disturbance can be formed as a colored noise model.

In terms of conditions a1 and a2, the simple controller cannot be satisfied, such as integral controller and proportional-integral (PI) controller. On the contrary, a number of calculations and stability analyses are required, when using a complex controller to meet a1 and a2. To condition a3, a large enough delay length is required according to the degree of disturbance, or it is unable to obtain an accurate result. However, the phase margin is decreased by the degree of delay, which degrades the performance of the controller, especially in high-speed applications. Towards condition b, it is hard to model the disturbance as a colored noise, because the disturbance of PZT is similar to white noise. Furthermore, the disturbance is unable to be measured individually, as it is generated by the inherent behaviors of PZT and the noise of sensors. Under these limitations, it is hard to adopt the method of adaptive noise canceling to filter the disturbance component in the output of the controller, where a reference signal related to disturbance must be provided. Therefore, an approach that presents the performance of a closed-loop system and easy implementation of online system identification is necessary. In terms of online system identification algorithm, as described in Section 1, a novel VFF-RLS should be proposed to reduce the effect of noise, which aims to estimate the reference model of PZT effectively and stably.

## 3. Design of Variable Forgetting Factor RLS Algorithm

In order to estimate the resonant frequency of PZT, the coefficients of reference model must be identified via system identification. According to (1), PZT nanopositioning system can be treated as a linear system. Thus, system identification based on the least squares principle can be adopted. Here, cost function can be defined as:

(3) $$J(k)=\sum_{i=0}^{k}e^{2}(i)=\sum_{i=0}^{k}{\left[d(i)-\hat{y}(i)\right]}^{2}=\sum_{i=0}^{k}{\left[d(i)-{\hat{\theta }}^{T}(i)\cdot x(i)\right]}^{2},$$

where e(i) is predict error, $\hat{y}(i)$ is predict output, d(i) is actual output, $\hat{\theta }(i)$ is weight vector and x(i) is data vector. The reference model of PZT can be obtained via updating the parameters of $\hat{\theta }(i)$ to minimize J(k). Then, estimation of PZT in real-time is required in this article, thus online system identification must be employed, where the RLS algorithm is widely used for this purpose. By recursively updating the parameters of $\hat{\theta }(i)$, the process of RLS algorithm can be described as:

(4) $$\hat{\theta }(k)=\hat{\theta }(k-1)+\Delta \hat{\theta },$$

where $\Delta \hat{\theta }$ is the correction. By modifying the $\hat{\theta }(k)$ in each circle, it aims to make e(k) tend to be zero. The detailed algorithm of RLS can be defined as:

(5) $$\{\begin{array}{l} K(k)=\frac{P(k-1)\cdot x(k)}{\lambda +x^{T}\cdot P(k-1)\cdot x(k)}\\ \hat{\theta }(k)=\hat{\theta }(k-1)+K(k)\cdot \left[d(k)-{\hat{\theta }}^{T}(k)\cdot x(k-1)\right]\\ P(k)=\frac{\left[I-K(k)\cdot x^{T}(k)]\cdot P(k-1\right)}{\lambda } \end{array},$$

where K(k) is Kalman gain, $P(k)={\left[x^{T}(k)\cdot x(k)\right]}^{-1}$ is the inverse of the input signal covariance matrix, and λ is the forgetting factor. The purpose of using λ is to overcome the data saturation phenomenon, where the performance of modification is reduced, and union error is increased by this phenomenon. By using λ, (3) is translated as:

(6) $$J(k)=\lambda \cdot J(k-1)+e^{2}(k),$$

where J(k) is formed as weighted summation. In order to demonstrate the forgetting property of (6), a memory time constant $T_{0}$ is defined in [25]:

(7) $$T_{0}=\frac{T_{s}}{1-\lambda },$$

where $T_{s}$ is the sampling time of the system. The measure data older than $T_{0}$ is weighed by less than 36% of the newest data. Therefore, the data saturation phenomenon can be eliminated by setting a suitable λ to reduce the effects of old data. As a result, the tracking performance of the algorithm can be ensured.

According to [26], the predict output can be approximated as $e(k)=\hat{y}(k)-y(k)$, when setting a low value to λ. Hence, e(k) can be described as disturbances, when λ≅1. Apparently, it can be indicated that $\hat{y}(k)≅y(k)$ is under this condition, which means the union error is zero. Thus, a value of λ close to one should be set to achieve an accurate result. However, for fast tracking, a low value of λ is desirable. Therefore, a constant λ is unable to cater to the requirements of both fast tracking and accurate estimation. In addition, the algorithm is more likely to be affected by disturbance, resulting from decreasing the weight of the old data. Thus, VFF is proposed to meet the above demands, where λ is replaced by a variable λ(k) in (5).

**Figure 2:**
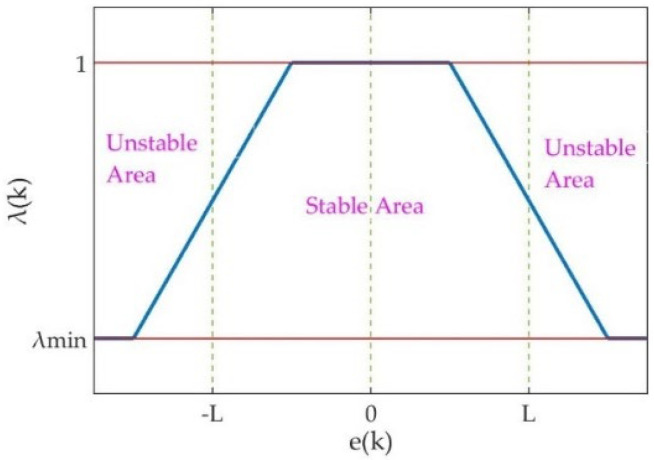
The ideal relationship between λ(k) and e(k).

In terms of identification result, the degree of deviation can be represented by e(k). Thus, λ(k) should vary according to e(k). A large value of λ(k) can be set to obtain stability and tracking accurately, which means the system is well identified. On the contrary, λ(k) can be reduced to ensure fast tracking. In addition, λ(k) is suggested to be placed between 0.95 and 0.99 in [27]. The ideal relationship between λ(k) and e(k) is plotted in Figure 2. λ(k) should be set close to 1, when e(k) within the boundary ±L. To achieve fast tracking, λ(k) is close to $\lambda_{min}$, where the $\lambda_{min}$ is the minimum of λ(k) to avoid the algorithm becoming unstable. However, e(k) fluctuates within different ranges, caused by the noise described as Section 2.3. Thus, a varying boundary is required to be adapted to the e(k), when it is desired to correlate λ(k) with e(k). Furthermore, modulus and sign of e(k) denote the degree and direction of deviation, respectively. Therefore, λ(k) is related to the modulus of e(k) via the above analysis. Here a varying boundary is defined as:

(8) $$L(k)=E(|e(k)|)=\frac{1}{k}\cdot \sum_{i=0}^{k}|e(i)|,$$

where the E(∣e(k)∣) is mean of ∣e(k)∣, and L(k) is absolute mean of e(k). 

L(k) is stable within a period of time, after e(k) starts to deviate the current range. It can be represented that the noise condition varies, when e(k) keeps on varying into a new range. Aims at this deviation, L(k) is modified to match the new range by (8). On the contrary, L(k) is maintained to hold the boundary. Thus, L(k) will be modified automatically to adapt to the latest range of e(k), which means the robustness to the noise. Furthermore, L(k) can be obtained at different holding time by using (6) and (7) to modify (8), according to the requirements. Herein, a relative error boundary is proposed, where the boundary is robust to the various ranges of noise. Then, an absolute deviation is defined as:

(9) $$E_{m}(k)=|e(k)|-L(k-1).$$

**Figure 3:**
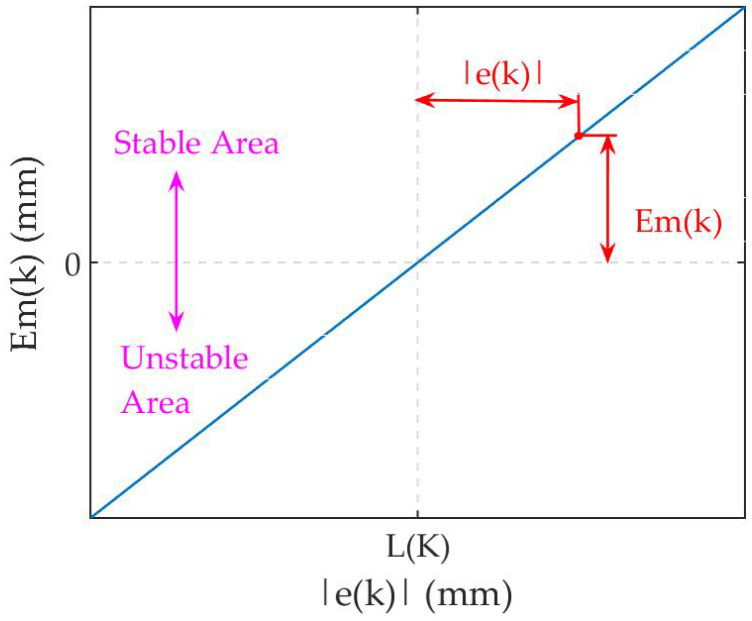
The relationship between $E_{m}(k)$ and ∣e(k)∣.

**Figure 4:**
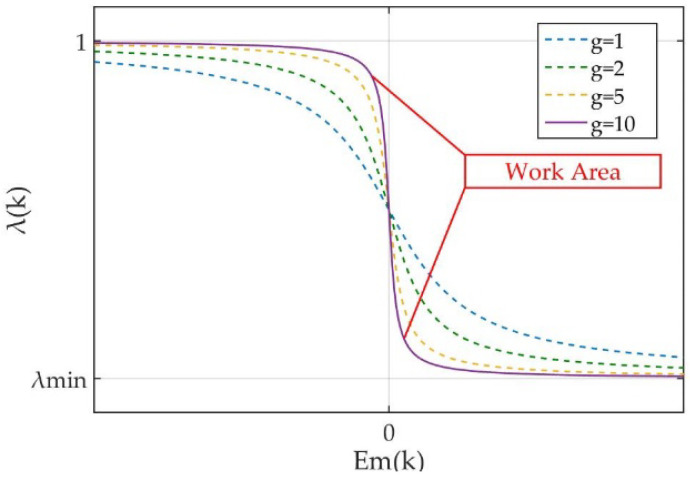
Image of varying forgetting factor function.

Figure 3 shows the relationship between $E_{m}(k)$ and ∣e(k)∣. Apparently, the zero of $E_{m}(k)$ is L(k). $E_{m}(k)$ is positive, when ∣e(k)∣ exceeds the boundary. This indicates that the deviation increases, which may result in an unstable system and need fast tracking. A negative value will be generated as ∣e(k)∣ is within the boundary, which denotes that stability is desired. The modulus of $E_{m}(k)$ represents the deviation degree between ∣e(k)∣ and L(k). Therefore, λ(k) should be varied according to $E_{m}(k)$. In order to correlate λ(k) with $E_{m}(k)$, the function expression of λ(k) is defined as:

(10) $$\lambda (k)=f_{a}\cdot \mathrm{acrtan}[g\cdot E_{m}(k)]+f_{0},$$

where g is the rate correction coefficient to determine the sensitivity of the algorithm to the identification error, $f_{a}=\frac{1-\lambda_{min}}{\pi }$ and $f_{0}=1-\frac{1-\lambda_{min}}{2}$. Figure 4 shows the function image of (10). Fast rate of varying will be obtained to λ(k) when in the work area. λ(k) is reduced when $E_{m}(k)$ is outside the work area. The width of work area is adjusted by g. A narrow width makes more sensitivity of λ(k) to e(k). λ(k) is close to 1, when e(k) places within boundary. On the contrary, λ(k) is close to $\lambda_{min}$. Herein, a novel VFF-RLS algorithm is proposed via the above design, which provides the performances both fast tracking and stability. It has the advantages of being insensitive to the noise and simple of implementation because only two parameters are required to be tuned.

## 4. Design of Position and Online Estimate Dual-Mode

In order to overcome the problem of implementation of closed-loop online system identification in PZT nanopositioning system, a novel structure of online estimation should be proposed. Since the input signal is not related to disturbance, implementation of open-loop system identification is much easier than closed-loop. However, the control performance of PZT is reduced due to the open-loop system. On the other hand, in terms of working conditions, the resonant frequency usually shifts under the conditions including pressurization and loading workpiece, etc. The motion is slow or static with vibrations, when conducted under the above working conditions. It takes time to stabilize the system after loading. Therefore, a frequency online estimate can be employed via the method of switching mode, where the effects of disturbance caused by mode switch are acceptable for the system under mass load. In order to implement the estimation, closed-loop system is switched to open-loop system. The steady-state error resulting from open-loop system identification can be eliminated via subsequent closed-loop feedback control. The advantages of easy utilization of open-loop system identification and good performances of closed-loop control can be combined by the approach of mode switch. However, this fluctuation generated by the mode switch may be unacceptable when used in some special applications. Generally, the fluctuation is acceptable, thus it is assumed in this article.

**Figure 5:**
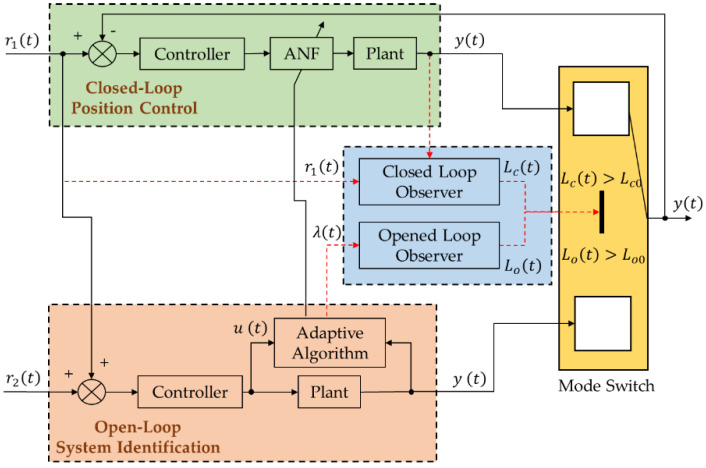
POEDM block diagram.

As shown in Figure 5, a control method called POEDM is proposed to implement online system identification in PZT nanopositioning system. POEDM consists of two modes, which are open-loop system identification and closed-loop position control. By respective observers, modes are switched and estimated the state. Reference signal tracking is carried out by feedback controller in closed-loop mode. The sharp resonance peak is eliminated to improve the closed-loop bandwidth by the ANF between controller and PZT. The state value of closed-loop observer is defined as $L_{c}(t)$ to compare with the closed-loop reference $L_{c0}$. $L_{c}(t)\geq L_{c0}$ indicates that the system is becoming unstable caused by the varying resonant frequency, when in closed-loop mode. Thus, for the implementation of online system identification, closed-loop mode is switched to open-loop mode. In order to achieve high accurate identification, an identification signal $r_{2}(t)$ is added to the reference tracking signal $r_{1}(t)$ according to the degree of disturbance, when performing open-loop system identification. The reference model, which is obtained by system identification, is used to update the $\omega_{n}$ of ANF. Similar to closed-loop mode, it can be determined that the system identification is completed, when the state value of the open-loop observer $L_{o}(t)$ is larger than the open-loop reference $L_{o0}$. When $L_{o}(t)\geq L_{o0}$, open-loop mode is switched to closed-loop to track the reference signal. Additionally, feedforward controller or compensation should be used to reduce the tracking errors of the open-loop system.

The following matters should be discussed:

1. The form of closed-loop reference $L_{c0}$ should be set according to the input reference signal. Under the stationary or slowly varying input signal, such as step signal, e(t) will not mutate immediately, when the resonant frequency of PZT is varying. In this case, $L_{c0}$ can be defined associate with $r_{1}(t)$, in terms of accurate tracking. Then, $L_{c0}$ can be set related to e(t), when input signal is non-stationary or fast varying signal, such as sinusoidal signal. To make it easier to observe the error, the logarithm of e(t) can be taken. In summary, $L_{c}(t)$ can be defined as:
  (11) $$L_{c}(t)=\{\begin{array}{l} a\cdot r_{1}(t),\;\mathrm{stationary}\;\mathrm{signal}\\ \log_{10}|e(k)|,\;\mathrm{non}-\mathrm{stationary}\;\mathrm{signal} \end{array},$$
  where a is the error range index and can be set from 1.05 to 1.10. Generally, the stationary signal is adopted in applications, thus the stationary $L_{c}(t)$ is assumed in this article.
2. The form of open-loop reference $L_{o0}$ should be defined associate with λ(k). It can be denoted that the identification is completed, when $\lambda (k)>(1-\lambda_{\min})/2$ and this will be demonstrated in Section 5.1.
3. Identification signal $r_{2}(t)$ for open-loop system identification is necessary. Due to the disturbance, union error exists in the result of system identification. Aims at reducing the error, an identification signal $r_{2}(t)$ is required. By using Gaussian noise in $r_{2}(t)$, accurate identification result can be obtained. The mean square error of $r_{2}(t)$ should be defined according to the degree of disturbance. It can be successful to implement identification without $r_{2}(t)$, when the frequency components of $r_{1}(t)$ is enough and the disturbances are not serious.
4. Minimum running time of closed-loop and open-loop mode $t_{c0}$ and $t_{o0}$ should be defined, respectively. The state values of mode $L_{c}(t)$ and $L_{o}(t)$ may be within their individual reference, caused by the fluctuation. Therefore, a long enough time is needed to ensure that the mode operates fully, where the system will become unstable due to frequent mode switch.

**Figure 6:**
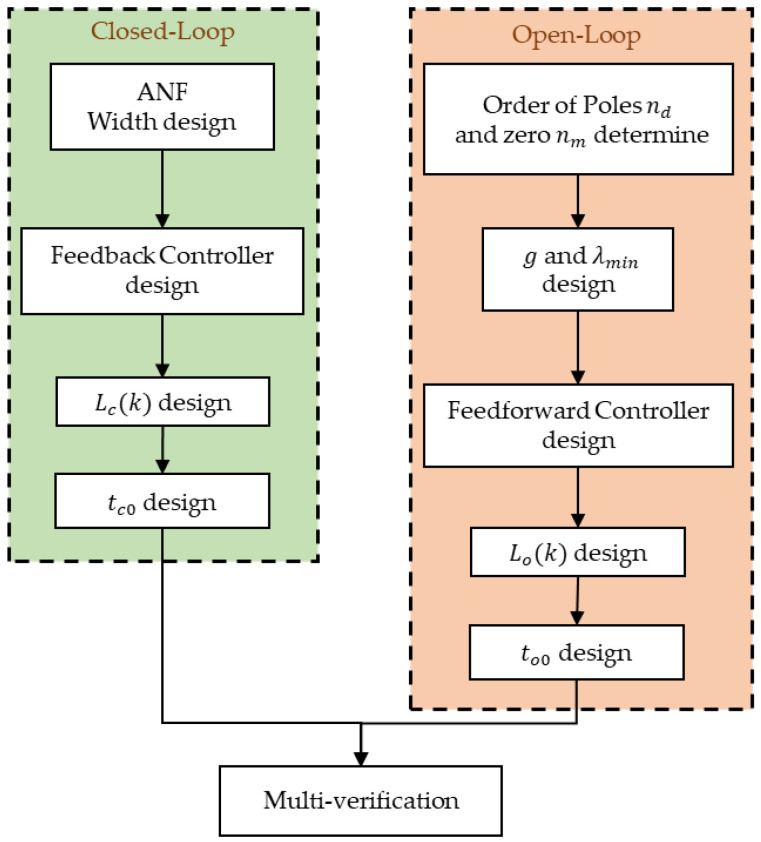
Implementation steps of POEDM.

The detailed implementation steps are illustrated in Figure 6. It should be mentioned that the step of model order determination can be carried out via the experimental approaches, including the cost function and F test. In terms of stability analysis, ANF can be treated as non-adaptive NF, because it is modified in open-loop mode, where the $\omega_{n}$ is unvarying in closed-loop mode. When using the LTI controller, the system can be characterized as being an LTI system, such as the PI controller in this article. Therefore, the tools including Bode diagram, Nyquist curve, and Routh criterion, can be used to analyze and design the controller according to the desired performances. If the stability of closed-loop mode is well designed, the tracking error generated by the open-loop mode can be eliminated by the feedback controller. According to (1), $n_{d}=2,\;n_{m}=0$ are defined in this article. The detailed control strategy is shown in Algorithm 1.
**Algorithm 1** Detailed control Strategy of POEDM.Design Variables: $n_{d},\;n_{m},\;\lambda_{min},\;g,\;a,\;L_{c0},\;L_{o0},\;t_{c0}\;\mathrm{and}\;t_{o0}.$
Initialization: $\begin{array}{l} \overset{⌢}{\theta }(0)=0_{1\times n},\;P(0)=\sigma \cdot I_{n},\;K(0)=0_{1\times n},\;\lambda (0)=1,\;t_{o}(0)=0,\;t_{c}(0)=0,\\ r_{s}(0)=0,\;L(0)=0\;\mathrm{and}\;sum(0)=0. \end{array}$
Nominal Values: $\begin{array}{l} n=n_{d}+n_{m}=\mathrm{elements}\;\mathrm{number}\;\mathrm{of}\;\mathrm{vector}\;x(k),\\ \sigma \in (10^{-9},10^{-6}). \end{array}$
$\begin{array}{l} \mathrm{Main}\;\mathrm{Loop}\text{:}\\ \mathrm{For}\;k=1:N\\ \;L_{c}(k)=a\cdot r_{1}(k)\\ \;e(k)=d(k)-x^{T}(k)\cdot \hat{\theta }(k-1)\\ \;L_{o}(k)=\lambda (k)\\ \;sum(k)=sum(k-1)+|e(k)|\\ \;L(k)=sum(k)/k\\ \;E_{m}(k)=|e(k)|-L(k)\\ \;\mathrm{If}\;t_{o}(k)<t_{o0}\\ \;t_{o}(k)=t_{o}(k-1)+1\\ \;r_{s}=1\\ \;\mathrm{End}\;\mathrm{If}\\ \;\mathrm{If}\;t_{o}(k)>t_{o0}\;\mathrm{and}\;L_{o}(k)>\frac{1+\lambda_{\min}}{2}\\ \;r_{s}=0\\ \;t_{c}(k)=t_{c}(k-1)+1\\ \;\mathrm{End}\;\mathrm{If}\\ \;\mathrm{If}\;t_{c}(k)>t_{c0}\;\mathrm{and}\;L_{c}(k)>L_{c0}\\ \;t_{o}(k)=0\\ \;t_{c}(k)=0\\ \;r_{s}=1\\ \;\mathrm{End}\;\mathrm{If}\\ \;\mathrm{If}\;r_{s}=1\\ \;K(k)=[P(k-1)\cdot x(k)]/[\lambda (k)+x^{T}\cdot P(k-1)\cdot x(k)]\\ \;\hat{\theta }(k)=\hat{\theta }(k-1)+K(k)\cdot \left[d(k)-{\hat{\theta }}^{T}(k)\cdot x(k-1)\right]\\ \;P(k)=[I-K(k)\cdot x^{T}(k)]\cdot P(k-1)/\lambda (k)\\ \;\lambda (k)=(1-\lambda_{\min})\cdot \arctan[g\cdot E_{m}(k)]/\pi +1-(1-\lambda_{\min})/2\\ \;\mathrm{End}\;\mathrm{If}\\ \mathrm{End}\;\mathrm{For} \end{array}$


## 5. Simulation Results and Discussion

**Figure 7:**
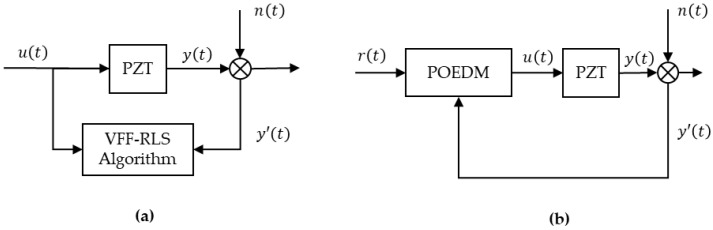
Block diagrams of (**a**) verification of VFF-RLS, (**b**) verification of POEDM.

The performances of POEDM are verified via a series of simulations in Matlab and Simulink. Since the PZT nanopositioning system is considered in the continuous-time domain, it needs to be discretized for the implementation of online system identification. By using bilinear z transformation, the translation function (1) is converted as follows:

(12) $$G(z)=\frac{b_{0}+b_{1}\cdot z^{-1}+b_{2}\cdot z^{-2}}{1+a_{1}\cdot z^{-1}+a_{2}\cdot z^{-2}},$$

where $z^{-1}$ is unit delay. The coefficients of (12) are calculated as:

$$\{\begin{array}{l} a_{1}=\frac{2\cdot \omega_{s}^{2}-2\cdot t_{a}^{2}}{d}\\ a_{2}=\frac{t_{a}^{2}-2\cdot \xi \cdot \omega_{s}\cdot t_{a}+\omega_{s}^{2}}{d}\\ b_{0}=\frac{\omega_{s}^{2}}{d}\\ \begin{array}{l} b_{1}=\frac{2\cdot \omega_{s}^{2}}{d}\\ b_{2}=\frac{\omega_{s}^{2}}{d}\\ d=t_{a}^{2}+2\cdot \xi \cdot \omega_{s}\cdot t_{a}+\omega_{s}^{2} \end{array} \end{array},$$

where $t_{a}=\frac{2}{t_{s}}$, and $t_{s}$ is the sampling time of the system. According to various of applications, $t_{s}=1\times 10^{-4}$ and ξ=0.01 are determined in this paper. As the disturbances can be treated as *n*(*t*) located after PZT in the verification of simulations, including inherent characteristics of PZT, noise of sensors and other factors, two structures of simulation are employed to be carried out and shown as Figure 7.

### 5.1. Variable Forgetting Factor RLS Algorithm Verification

First, the performances of proposed VFF-RLS algorithm are verified. As demonstrated in Figure 7a, by employing the method of open-loop online system identification, signal of $u(t)=r_{1}(t)+r_{2}(t)$ is inputted to (12) via different algorithms. The input reference signal used in applications can be described as step, sinusoid and ramp or combination of them. Thus, $r_{1}(t)$ is defined as above signals with unit amplitude, respectively. The fundamental frequency of sinusoid is 1 Hz. The step and ramp signals vary at the 1st second. $r_{2}(t)$ is set as unit mean square error Gauss noise for ease of evaluation. The simulations last 6 s and the resonant frequency of PZT varies from 56 Hz to 40 Hz at the 2nd second, which purposes to simulate the process of mass load. Various levels of Gauss noise are added to the output to simulate disturbance, where the levels of the noise are defined as signal-to-noise ratio (SNR). Compared with RLS and FF-RLS algorithm, the performance of varying resonant frequency online estimate of VFF-RLS algorithm is verified, where the forgetting factor of FF-RLS is defined as $\lambda_{FF}=(1-\lambda_{min})/2$.

**Figure 8:**
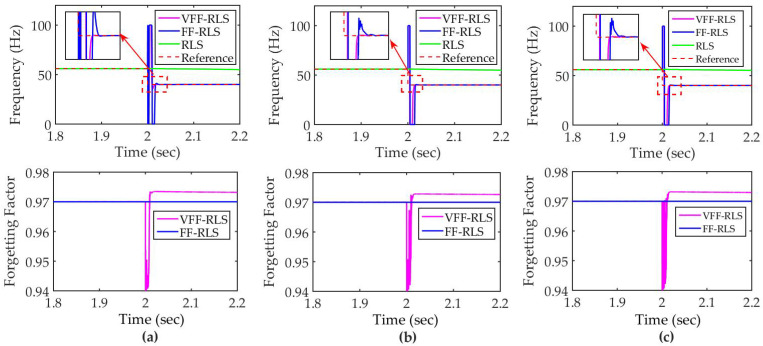
Simulation results of frequency estimate and forgetting factor under SNR = 60 dB: (**a**) sinusoid; (**b**) step; (**c**) ramp.

Figure 8 shows the results of frequency estimation with different algorithms. Apparently, due to the data saturation phenomenon, RLS is unable to track the varying frequency. λ(k) of VFF-RLS is lower than $\lambda_{FF}$, when the frequency is starting to vary. As result, VFF-RLS tracks the frequency faster than FF-RLS. λ(k) is larger than $\lambda_{FF}$, after the algorithm convergences, which purposes to obtain stability. In order the analyze the results in more detail, two indicators are defined to evaluate the performances. First, mean square error (MSE) is described as:

(13) $$\mathrm{MSE}=\frac{\sum_{i-0}^{k}e^{2}(i)}{k}=\frac{\sum_{i-0}^{k}{\left[y(i)-\hat{y}(i)\right]}^{2}}{k},$$

where the MSE indicates the degree of overall estimate error. Then, settle time (ST, second) is defined to describe the first time it takes to reach the reference frequency and place within ±105% of the frequency, after it varies to 40 Hz. The detailed results are recorded in Table 1. Compared with FF-RLS, the ST of VFF-RLS is reduced by nearly 50%, while the RLS is unstable. In terms of overall error, the MSE of VFF-RLS is 80~90% of FF-RLS. It demonstrates that the proposed VFF-RLS algorithm has the advantages of both fast tracking and stability. Furthermore, discussion 2 in Section 5 can be easily proved by Figure 8, where $\lambda (k)>(1-\lambda_{\min})/2$ denotes that the estimation has been completed.

### 5.2. Position and Online Estimate Dual-Mode Verification

**Figure 9:**
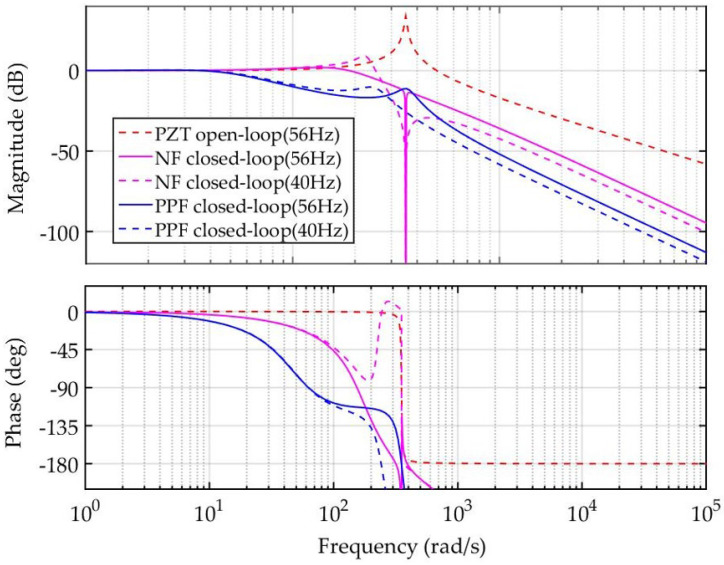
Comparison of Bode diagram with different approaches.

Herein, a series of simulations are carried out to verify the performances of POEDM, where the structure is plotted in Figure 7b. Aiming at demonstrating the superiority of gain margin improvement of ANF, positive position feedback (PPF) control and NF are used as control group. The frequency of PPF and NF is defined as 56 Hz and other parameters are set to obtain optimal performances. For ease of analysis, unit gain proportional controller is employed. The Bode diagrams are plotted under the resonant frequency of PZT nanopositioning system at 56 Hz and 40 Hz, respectively. The result of Bode diagram is shown as Figure 9. It indicates that the gain margin improvement of NF is better than PPF, before the resonant frequency varies. However, the gain margin of NF drops to be negative after the frequency varies, while the decrease in PPF is acceptable. According to the above analyses, better gain margin can be ensured by using ANF, which can track the varying resonant frequency in real-time.

In order to verify the performance of POEDM, simulation of frequency online estimate is conducted, compared with open-loop and closed-loop system identification. The resonant frequency of PZT varies from 56 Hz to 40 Hz at the 6st second. Gauss noise is adopted as input signal, and the SNR is 40 dB.

**Figure 10:**
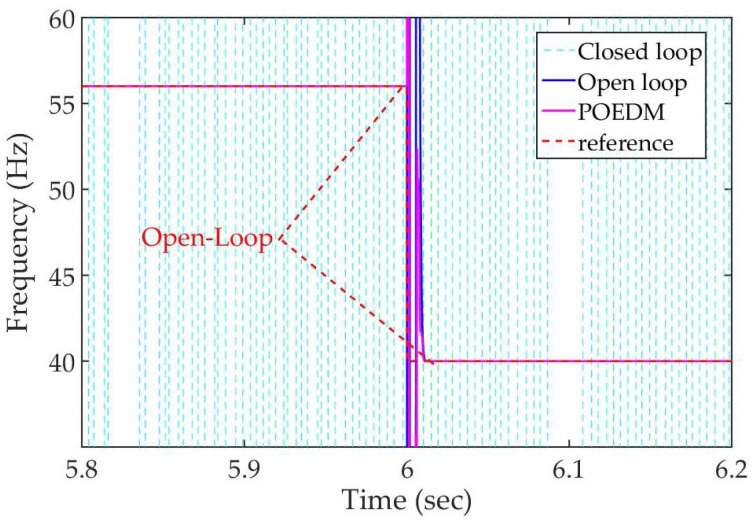
Comparison of frequency estimate under SNR = 40 dB.

The result is illustrated in Figure 10. The closed-loop fails to identify, while the tracking performance of POEDM is similar to open-loop system identification. Other conditions including SNR = 30, 50 and 60 dB show the same result, thus they are omitted in this paper.

To verify the tracking performance of POEDM, simulations are implemented by using triangular trajectories with fundamental frequencies of 1, 2, 3, 5, 10 and 30 Hz. Due to the advantages of simple implementation and robustness to modeling errors, proportional-integral (PI) controller has been wildly used in various applications [28]. Therefore, PI is adopted to be the feedback controller in these simulations. Compared with PF and NF, the tracking performance of POEDM is proved, where the PI is adjusted to achieve the optimal parameters, respectively. The resonant frequency of PZT varies from 56 Hz to 40 Hz at the 6st second. The simulations last 20 s with a Gauss noise as disturbance, where the mean square errors of Gauss noise is $\sigma_{n}^{2}=10^{-6}$. Due to the linear model of PZT nanopostitioning system, the performance of simulations is the same under different scales. Therefore, in order to easily analyze, millimeter-scale of displacement (Disp) is adopted in simulations, while the precision of applications is nanoscale. In terms of tracking performances analysis, perfectly delayed tracking should be employed instead of imperfect timely tracking, when the delay is well known [5]. Thus, the following two indexes are used in this work, according to [29]:

(14) $$e_{m}=\frac{\underset{t\in [0,T]}{\max}|y(t)-r(t-k^{*}\cdot t_{s})|}{\max|r(t)-\min(r(t))|}\times 100\%,$$

(15) $$e_{rms}=\frac{\sqrt{\frac{1}{T}\cdot \int_{0}^{T}{\left[y(t)-r(t-k^{*}\cdot t_{s})\right]}^{2}dt}}{\max[r(t)-\min(r(t))]}\times 100\%,$$

where the $e_{m}$ and $e_{rms}$ are the maximum error and the root mean square error, respectively. T is the fundamental frequency of reference signal. *r*(*t*) and $r(t-k^{*}\cdot t_{s})$ are the reference signal and shifted reference signal, respectively. The $k^{*}$ in (14) and (15) is defined as:

(16) $$k^{*}=\arg_{k}\min[\underset{t\in [0,N\cdot T)}{\max}|y(t)-r(t-k\cdot t_{s})|],$$

where *k* is a variable value defined within $\left[0,\;T/T_{s}\right]$. Because the error of tracking lag can be eliminated via compensation or other control methods, this lag is not included in (14) and (15). The approaches toward the lag are not discussed in this paper.

**Figure 11:**
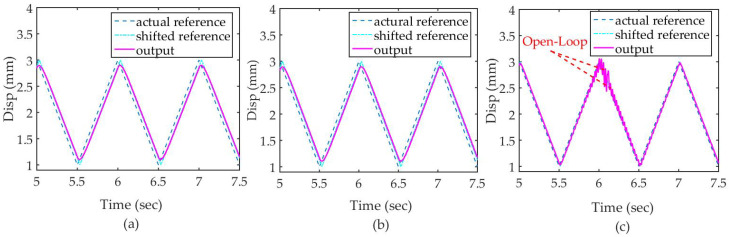
Simulation result under 1 Hz: (**a**) PI with PPF; (**b**) PI with NF; (**c**) PI with POEDM.

As shown in Figure 11, the lag of POEDM is less than others, when the input frequency is 1 Hz. Due to the open-loop mode, the output of POEDM fluctuates at the 6st second, which is stabilized by the closed-loop mode subsequently.

According to (14), tracking error is defined as:

(17) $$\mathrm{Tracking}\;\mathrm{error}=y(t)-r(t-k^{*}\cdot t_{s}).$$

**Figure 12:**
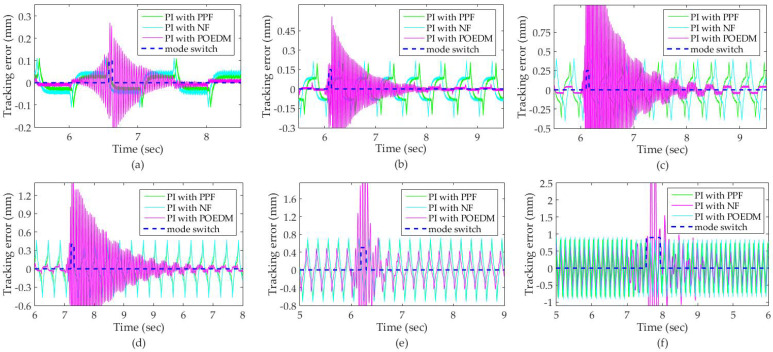
Tracking error under the input frequency of: (**a**) 1 Hz; (**b**) 2 Hz; (**c**) 3 Hz; (**d**) 5 Hz; (**e**) 10 Hz; (**f**) 30 Hz.

The performance of tracking reference signal is evaluated by (17) and its results are plotted in Figure 12. The dot-line in blue denotes the state of the mode switch, where the falling and rising indicate the closed-loop and open-loop, respectively. According to Figure 12, the performance of PPF and NF decreases seriously as the input frequency decreases, compared with POEDM. Because the closed-loop reference $L_{c0}$ is defined associated with the $r_{1}(t)$, the mode has changed to open-loop after the system started to become unstable but not the resonant frequency varied. The fluctuation generated by open-loop mode is eliminated after switching to closed-loop mode. It should be noted that enough $t_{c0}$ is necessary, or the system will become unstable due to frequent mode switch. Because the stable system works in closed-loop mode, $e_{m-cl}$ is defined as the max tracking error of POEDM, where $e_{m}$ is replaced by $e_{m-cl}$.

The results of tracking error are recorded in Table 2. It can be noted that $e_{rms}$ of POEDM is reduced by 39.5% and 50.5%, respectively, when under 5 Hz and compared with PPF and NF. Meanwhile, $e_{m-cl}$ is reduced by 83.8% and 85.1%, respectively. The $e_{rms}$ and $e_{m-cl}$ of POEDM are kept within 5% and 4% when the input frequency is lower than 5 Hz. It should be noted that both of them exceed 5% as the frequency is over 5 Hz. Thus, the performance of POEDM decreases severely when the input frequency is higher than 5 Hz. According to the knowledge of signal processing, the triangular waveform can be approximated by its fourth odd harmonics [5]. Therefore, the bandwidth of the closed-loop system is 35 Hz when POEDM is employed. It should be concluded that the input signal and the bandwidth are achieved up to 12.5% and 87.5% of the resonant frequency of the PZT nanopositioning system, respectively.

As shown in Table 3, the minimum running time of open-loop mode and closed-loop mode is denoted as $t_{c0}$ and $t_{o0}$, respectively. The actual duration of them is represented as actual *t_c_* and actual *t_o_*. It can be noted that, except for the simulation of 30 Hz, the open-loop online system identification is completed within $t_{o0}$.

## 6. Conclusions

In order to realize the varying resonant frequency online estimate and reducing the effect of the lightly damped resonance, ANF for PZT nanopositioning system via POEDM was proposed in this paper. Besides, a novel VFF-RLS algorithm was demonstrated for system online identification, which has the advantages of strong robustness, easy implementation and superior tracking performance. Furthermore, a POEDM approach was developed to estimate the varying resonant frequency in the presence of noise and disturbance. Finally, a series of validation simulations were successfully carried out and the results indicate that the proposed VFF-RLS algorithm is nearly 50% fast than FF-RLS in the frequency tracking and has good stability, under various conditions. The performance of POEDM online estimation is similar to open-loop system online identification, while closed-loop failed to identify. The max error and root mean error of POEDM can be kept within 5% under the input frequency below 5 Hz, while PPF and NF decrease severely as the input frequency increases. In summary, by employing the control method of PI with POEDM, the input signal frequency and closed-loop bandwidth are increased to 12.5% and 87.5% of the resonant frequency, respectively. However, this work is considered that PZT works within the linear area, while the nonlinear behaviors limit the performances in applications. About 10% of the bandwidth is within the nonlinear area, which will deteriorate. In order to present higher performances, future work should focus on overcoming the effect of nonlinear characteristics.

## Figures and Tables

**Table 1:** Comparison of different signal under different SNR.

Signal	SNR (dB)	RLS		FF-RLS		VFF-RLS	
		ST (sec)	MSE	ST (sec)	MSE	ST (sec)	MSE
Sinusoid	40	N/A	7.397	2.048	0.43	2.028	0.368
	50	N/A	7.28	2.042	0.285	2.021	0.226
	60	N/A	7.244	2.031	0.24	2.018	0.181
Step	40	N/A	7.236	2.034	0.465	2.015	0.42
	50	N/A	7.156	2.032	0.27	2.014	0.237
	60	N/A	7.13	2.031	0.221	2.014	0.189
Ramp	40	N/A	7.28	2.045	0.385	2.017	0.355
	50	N/A	7.205	2.035	0.26	2.016	0.231
	60	N/A	7.182	2.028	0.221	2.016	0.192

**Table 2:** Comparison of simulation result.

Signal (Hz)	PI with PPF		PI with NF		PI with POEDM		
	*e_m_* (%)	*e_rms_* (%)	*e_m_* (%)	*e_rms_* (%)	*e_m_* (%)	*e_m-cl_* (%)	*e_rms_* (%)
1	5.5	1.5	5.8	2.1	13.3	1.5	1.1
2	10.1	3.1	10.8	4.6	28.2	1.9	2.5
5	22.2	7.6	24.1	9.3	85.2	3.6	4.6
10	34.0	14.2	35.5	15.7	143.5	23.5	11.2
30	41.5	19.2	45.8	20.5	225.0	36.0	18.2

**Table 3:** Minimum running time of the simulations in Figure 12.

Signal (Hz)	PI with POEDMC			
	*t_c_*_0_ (sec)	Actual *t_c_* (sec)	*t_o_*_0_ (sec)	Actual *t_o_* (sec)
1	2	6.68	0.05	0.05
2	2	6.08	0.05	0.05
3	2	6.1	0.05	0.05
5	2	6.11	0.05	0.05
10	3	6.09	0.05	0.05
30	3	6.14	0.1	0.76

## Data Availability

Data available on request due to privacy. The data presented in this study are available on request from the corresponding author. The data are not publicly available due to the future work closely related to the subject of this article.
